# Beans

**Published:** 1889-11

**Authors:** 


### BEANS.

It is the fashion nowadays to descry this staple food of our grandmothers. Beans are said to be coarse, indigestible, only suitable for the laboring classes. It is even whispered at times that they are vulgar, and when Madam Grundy issues this edict, who so bold as to defy and persist? Let us be deceitful, let us be vain, nay, even let us be dishonest, but vulgar!—shades of our ancestors—never! Therefore I propose to put in a plea for this same despised bean—to maintain, and endeavor to prove, that they are more sinned against than sinning, that properly cooked and served, they form a most nutritious, appetizing, healthful and economical food, not only for stout men and boys, but for delicate women and children as well. Not one time in a hundred are they properly cooked, especially when left to servants. They contain 24 per cent. of nitrogenous matter in the form of legumine, or vegetable caseine, and are therefor more highly nutritious than almost any food. Were it not for the fact that, as usually cooked, they are rather more difficult of digestion than many other foods, there would be no question as to their supereminence as a diet. One pound of beans contains nearly six ounces of heat-producing properties and half an ounce of flesh-forming food, which is more than twice as much of the flesh-food, and nearly as much of the heat food as wheat contains.

